# Semi-supervised Learning for the BioNLP Gene Regulation Network

**DOI:** 10.1186/1471-2105-16-S10-S4

**Published:** 2015-07-13

**Authors:** Thomas Provoost, Marie-Francine Moens

**Affiliations:** 1 Computer Science Department, KU Leuven, Celestijnenlaan 200A, 3001 Heverlee, Belgium

**Keywords:** Machine Learning, Relation Learning, Semi-supervised

## Abstract

**Background:**

The BioNLP Gene Regulation Task has attracted a diverse collection of submissions showcasing state-of-the-art systems. However, a principal challenge remains in obtaining a significant amount of recall. We argue that this is an important quality for Information Extraction tasks in this field. We propose a semi-supervised framework, leveraging a large corpus of unannotated data available to us. In this framework, the annotated data is used to find plausible candidates for positive data points, which are included in the machine learning process. As this is a method principally designed for gaining recall, we further explore additional methods to improve precision on top of this. These are: weighted regularisation in the SVM framework, and filtering out unlabelled examples based on a probabilistic rule-finding method. The latter method also allows us to add candidates for negatives from unlabelled data, a method not viable in the unfiltered approach.

**Results:**

We replicate one of the original participant systems, and modify it to incorporate our methods. This allows us to test the extent of our proposed methods by applying them to the GRN task data. We find a considerable improvement in recall compared to the baseline system. We also investigate the evaluation metrics and find several mechanisms explaining a bias towards precision. Furthermore, these findings uncover an intricate precision-recall interaction, depriving recall of its habitual immediacy seen in traditional machine learning set-ups.

**Conclusion:**

Our contributions are twofold:

1. An exploration of a novel semi-supervised pipeline. We have succeeded in employing additional knowledge through adding unannotated data points, while responding to the inherent noise of this method by imposing an automated, rule-based pre-selection step.

2. A thorough analysis of the evaluation procedure in the Gene Regulation Shared Task. We have performed an in depth inquiry of the Slot Error Rate, responding to arguments that lead to some design choices of this task. We have furthermore uncovered complexities in the interplay of precision and recall that negate the customary behaviour commonplace to the machine learning engineer.

## Background

The set of BioNLP shared tasks [1] form a biannual challenge used by many to apply and develop state-of-the-art methods in the field of biomedical information extraction (IE). In 2013 in its third instalment, it again succeeded in attracting a considerable amount of contributions from an international community of researchers. This work is spread over six different subtasks, each with a focus on fine-grained IE to construct knowledge bases in their respective domain.

**Figure 1:**
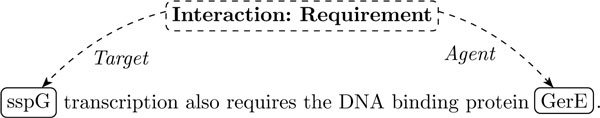
**Example sentence from task data set: there is an Interaction:Requirement relation defined between entities GerE and sspG**. Entity annotations (full-line border) are given in the test phase. The relation type (here: Interaction:Requirement) and which entities are target and agent for the relation need to be predicted.

The Gene Regulation Network subtask [2] tries to attain the construction of a relation network encompassing the extracted knowledge, in order to build models to represent the behaviour of a system. This network can then serve as a base for representing current knowledge, and be leveraged for making inferences and predictions, i.e. towards experiment design. In the case of this particular task, this system entails the whole of molecular interactions between genes and proteins in a specific bacterium, the *bacillus subtilis*. An example sentence for this task is given in Figure 1.

**Figure 2:**
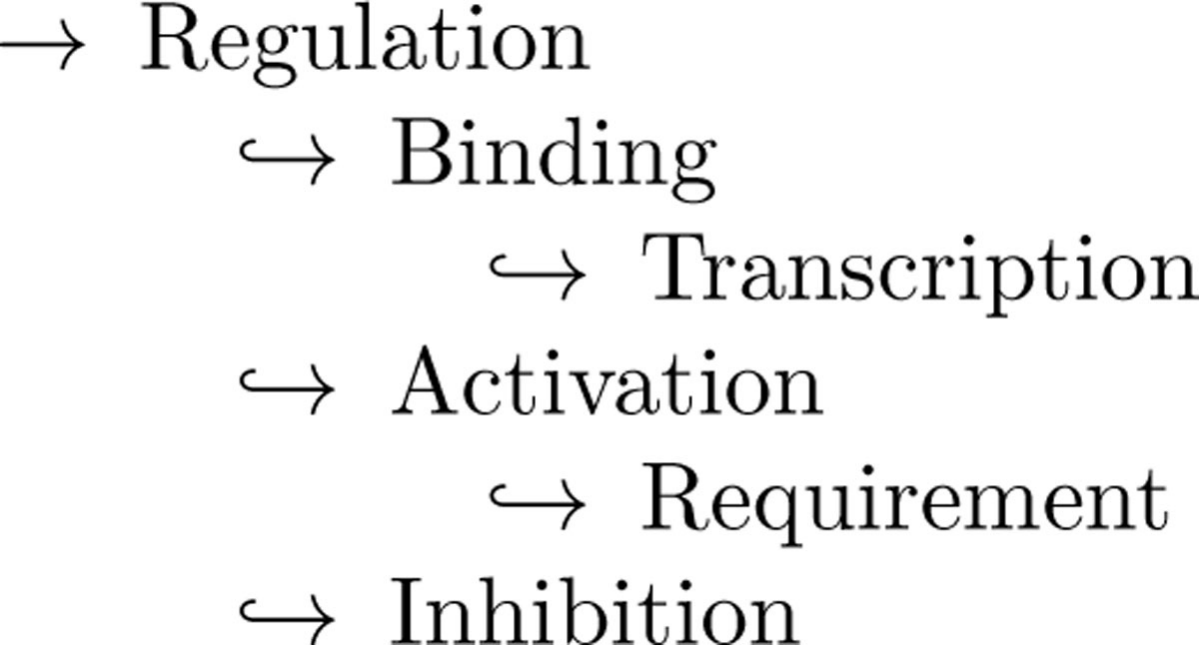
**Hierarchy imposed on the output types of the GRN task**.

Participants are asked to extract a regulation network from sentences taken from PubMed abstracts describing these phenomena. This network is comprised of six different types of relations, which are related into a small hierarchy (see Figure 2). At both train and test time, gold standard annotations of entities are provided, making this a pure relation extraction task, without the need to do named entity recognition, a task with its own set of difficulties and challenges. Of further note is the fact that submissions are evaluated on the produced network as a whole, namely the set of relations detected on the test data as a whole. We discuss the impact of this *global *scoring in the section Results and discussion. In the systems produced for this task, we notice a strong tendency to favour *precision*, i.e. controlling the false positive rate. The top submission [3] obtained a precision score of 68%, however only reaching a recall of 34%. While there certainly is a need for reliable results when working with biomedical knowledge, covering a sufficient proportion of true positives (i.e. *recall*) can be equally fundamental in many practical applications. Examples of these are hypothesis generation and knowledge base construction, especially in settings where adding more data can not solve the problem of finding additional true positives (as can be the case in e.g. texts describing recent findings). Indeed, the interest in developing systems for inference and/or prediction equally lies in the retrieval of a sizeable hypothesis set, rather than reaching only those that can be found with high confidence. One way to balance a system in favour of recall is the exploitation of additional unannotated data. By working in a semi-supervised fashion, a learner can be made more aware of the wide variety of patterns encoding a relationship. This happens at the cost of introducing more noise (and hence decreasing precision), since there is no reliable way of labelling the extra data. In this paper we explore a method to decrease this cost, effectively keeping precision stable while improving recall.

Basing ourselves on the model of [4], that achieved a second place for this task, we explore how semi-supervised techniques can improve the performance that this system obtains in its supervised form. We further investigate several techniques to counterbalance the noise added by these methods. Next to the traditional measure of weighing regularisation parameters, we go on to develop a novel method based on probabilistic rule-finding. Next, we look at the experimental set-up and compare the results of the proposed methods. We also discuss some of the properties of this task, and evaluate how these can impact performance in terms of precision and recall. This influence can be both direct, e.g. because of data skewness or pre-scoring processing, and indirect. An example of the latter is found in the choice of the final scoring metric (the Slot Error Rate), altering some of the parameter choices when designing and selecting a model.

The section thereafter reviews related work. We finish with conclusions and future research questions.

## Methods

### Baseline model

We base ourselves on the model of [4]. The main reasons for this are as follows:

• Their model came in second place, showing decent performance;

• Unlike the winning entry, their model does not use hand-crafted rules, and is based on Support Vector Machines. Their set-up therefore lends itself perfectly to extension into a semi-supervised framework as described below.

The main configuration of the system of [4] is a collection of Support Vector Machines (SVMs, see [5]), one per relation type. The authors construct a data point for each couple of genic entities in a sentence, effectively considering all potential agent/target pairs for the relations. The kernel used is a Gaussian RBF kernel (see [6] for the seminal work, and [7] for a good overview).

The novelty of [4] lies in the feature construction. The feature vectors for candidate relation tuples are built as follows:

$$f=f_{base}⊙f_{context}$$

This is a concatenation (symbolised by ^©^) of *local *features *f_base_*, complemented by what is referred to as *context *features, *f_context_*. The *local *features consist of the classical word information (stem + part-of-speech) along with the biomedical compound type (e.g. *Gene, Protein*) for the words that the entities comprise, with different parts for the agent and target entities. The *context *part is then constructed in the following fashion, also separately for both entities:

$$f_{context}\left(\omega \right)=\frac{1}{z}\underset{w_{i}\in sentence}{\sum }\alpha^{d\left(w,w_{i}\right)}f_{base}\left(\omega_{i}\right)$$

with *w *being the words of the entities at hand, and the sum going over all the *w_i _*words in the sentence. *d(w, w_i_) *is the distance in number of words between *w *and *w_i_*. This is in essence an average of the vectors encoding the different non-entity words in the sentence, weighted inversely by their distance to the entity words. *α* is a constant controlling how fast the weights decay with distance, and *Z *is a normalisation factor. Note that the traditional fashion of including textual context exists of *concatenating *these separate word vectors instead of averaging. This leads to feature vectors with only values of 0 or 1 as components, whereas the entries in *f_context _*can take on all real values in the interval [0,1]. We direct the reader to the work of [4] for further details.

A few specific differences are to be noted between our implementation and that of the submitted system. We use the LibSVM [8] package as provided by the Scikit-learn Toolbox [9]; this difference in library used should be of minor influence on results, and we are indeed able to replicate their performance. Furthermore, as mentioned in the original paper, the distance *d *used for the submitted results was taken to be the distance in the parse tree, where later tests proved to be more favourable towards using a 'flat' sentence distance, as described above. We compared both options in a cross-validation setting (utilising trees generated from the parser by [10]) and found indeed the use of the latter to give better results. We use a value of *α *= 0.9.

### A distant learning approach

The main issue of a fully supervised system is the difficulty to generalise towards unseen patterns. This problem is more apparent the sparser the data, and the richer the representation. With our baseline system having an elaborate feature representation, we suspect this to be a big factor in this framework. Furthermore, new data points will likely entail unseen words, in part counterbalancing the effectiveness of this sort of feature scheme, albeit widely used in NLP situations (as shown in e.g. [11] and [12]). Because of these reasons, the base system is likely to suffer from a poor generalisability, as also testified by its poor recall score.

A corpus of related, but unseen data points can provide a source of new patterns to incorporate in our learner. Of course, the main obstacle is the lack of labelling for this data; we have no knowledge what points are to be marked as positive. Instrumental in any semi-supervised framework are therefore:

• An approximation method to identify the labelling of unseen data. As this can never fully substitute the precision of annotations supplied by a human expert, the uncertainty in this introduces additional noise. Hence also the need for the next item:

• Means of managing the uncertainty in adding unlabelled data. Since the labellings now contain more noise, this inherently changes the optimal learning strategy; a semi-supervised method needs to take this into account.

We propose an expansion to the distant supervision framework (see [13,14]). In this line of methods, the classifier is trained on a set of 'bags' of data points, with the defining property that positive bags are only known to be partly containing positively labelled points. The negative bags on the other hand are more certain to effectively contain only negative points. As shown in [14,15], one use case for this set-up is exactly relation learning, in the event of having a set of known relations between two entities, but when no finer-grained annotations (i.e. on a document or sentence level) are available.

Contrary to this framework, we do have at our disposal the fine-grained annotations of our labelled data set. However, the structure of these distant learning problems points us to the aforementioned approximation method to add unlabelled data to the training data. Namely, the following observation is used: if a biological relation exists between two entities (as seen in the labelled data), there is a substantial probability that another (unlabelled) sentence containing both entities will also encode this relation. We therefore add any data point from the unannotated corpus that is composed of two such entities to the training set, labelling it as positive. Note that, since our main goal is to introduce new patterns to the classifier, we also use the vocabulary from these sentences when constructing feature vectors. This ensures that we use an unbiased representation of these data points.

Opposite to the case of positive examples, the same inference can not be performed here to extract negative data points. Absence from a sparse set of known relations only marginally changes the probabilities on these points. We therefore refrain from adding negatives from the unlabelled data, barring further methods to obtain a more accurate selection. This is where our case differs from most distant supervision systems, who are able to extract negative data points due to either explicitly providing negative seed examples, or having ample data to employ a *closed world assumption *[16]. The latter presumes an adequate coverage of positive data, such that everything outside of this knowledge is seen as negative. As will be seen, the pre-selection filter we develop in the following subsection provides us with an alternative method to extract negatives; there we will revisit our choice.

We will refer to the above method as the 'basic' method (cfr. in results Table 1 the entry [BASIC]), as opposed to the systems augmented with the techniques described below.

**Table 1:** Comparative table of results for our different systems.

System	S	D	I	C	M	Recall	**Prec**.	F1	SER
Original submission of [4]	15	53	5	20	40	22.7	50.0	31.3	0.830
[BASIC]	28	18	100	42	170	47.7	24.7	32.6	1.659
[W_REG]	30	12	204	46	280	52.3	16.4	25.0	2.795
[PRE_SEL]									
select POS, no NEG	28	20	77	40	145	45.5	27.6	34.3	1.420
select POS, select NEG	13	39	21	36	70	40.9	51.4	45.6	0.830
select POS, all NEG	13	43	22	32	67	36.4	47.8	41.3	0.886

All results are obtained on the official test set. (*S *= substitutions, *I *= insertions, *D *= deletions, *C *= correct predictions, *M *= number of predictions, *N *= 88 = number of arcs in reference network).

### Methods of counterbalancing the added noise

Whenever reliability of labelling is affected, this directly influences precision. The basic method proposed above is guaranteed to introduce new patterns to the classifier, which is expected to improve recall. However, this comes at the cost of adding uncertainty to the labelling of the data, which is prone to an increase in false positives.

In this part, we will look at different methods to counter this effect and maintain adequate precision. We study the effects of a general method known to deal with different kinds of noise, namely having a non-constant regularisation parameter in the SVM. We then move on to develop a method of pre-selecting the data that is added from the unlabelled corpus, leading to a more fine-grained control of the introduced uncertainty.

#### Weighted regularisation

A conventional way to deal with noisy training examples comes with the observation that, in the traditional set-up, only the positive data points are plagued by this noise. Hence, in a soft-margin SVM framework (as developed by [17]), a different regularisation policy is introduced for positive and negative examples, as first proposed by [18], and later also employed by e.g. [15,19]. Let χ^+^, χ^− ^be the set of positive and negative data points respectively, and *ϕ*(*x*) be the feature representation for *x*, this then leads to the following optimisation formulation:

$$\underset{w,b,\xi_{x}}{\text{min}}\left(\frac{1}{2}||w|{|}^{2},+,c^{+},\underset{x\in \chi^{+}}{\sum },\xi_{x},+,c^{-},\underset{x\in \chi^{-}}{\sum },\xi_{x}\right)$$

subject to:

$$\begin{array}{ccc} \left\langle w,\phi \left(x\right)\right\rangle +b\geq & 1-\xi_{x}, & \forall_{x}\in \chi^{+}\\ \left\langle w,\phi \left(x\right)\right\rangle +b\leq & -1+\xi_{x}, & \forall_{x}\in \chi^{-}\\ & \xi_{x}\geq 0, & \forall_{x}\in \chi^{+}\cup \chi^{-} \end{array}$$

*w *is the weight vector that defines the separating hyperplane together with the constant *b *as a bias term. The $\xi_{x}$ serve in this optimisation problem as *slack variables*, allowing a trade-off of maximising the margin against having a few points surpassing that margin. By having two regularisation constants *C*^+ ^and *C*^− ^we can allow the margin for positive points to be 'softer', accounting for the additional uncertainty in this subset.

#### An automatic rule-detection algorithm for pre-selection of unannotated data

Many machine learning systems that serve a specific application make use of a framework that incorporates specialist knowledge. A prevalent mechanism for this is by having some rule-based pre-/post-processing. We propose a method for extracting some of this knowledge from the labelled data in a fully automated fashion. This mechanism covers many standard techniques regularly used by system engineers, such as filtering on trigger words that explicitly refer to interactions ('transcription', 'binding', ...) [16,3], or on the type of bio-molecule for specific roles (e.g. the target of a *Binding *event is a *Gene *or *Site *entity) [2,3]. However, the automatic nature of our method discards the need for manually identifying and pinpointing useful rules. Furthermore, it is agnostic of the nature of the data, and hence perfectly adaptable to texts in any domain or task.

In the framework of our semi-supervised system, this can then be used to obtain a more fine-grained selection from our unlabelled corpus. We do this by extracting patterns from the features of the labelled training data, and including from the unannotated data only those points that also adhere to these observed patterns.

As we are dealing with a pre-selection step on what is expected to be positive, our main focus is on detecting sufficient conditions in the feature space for negativity. In order to find such a rule implicitly present in the data, we observe the following:

$$\begin{array}{ccc} \left(f_{i}\in V_{i}\to 0\right) & \Rightarrow & \left(1\to f_{i}\notin V_{i}\right)\\ & \Rightarrow & P\left(f_{i}\in V_{i}|1\right)=0 \end{array}$$

where *f_i _*is the *i*th feature of a data point, *Vi *a set of values, and 0,1 have been used as shorthand for the (negative resp. positive) labelling of that point. The extension towards rules that conjoin several features is immediate.

While the above observation is necessary for a negative labelling, it is by no means sufficient, i.e. finding a zero frequency can not exclude chance, especially in small datasets. To see how much of a factor *f_i _*effectively is in the labelling of the point, one could look at probabilistic measures such as Mutual Information, Bayes Factor or the Kullback-Leibler divergence. However, most of these measures are only meaningful on non-zero probabilities, mainly because of the occurrence of logarithms or divisions of these probabilities.

To escape the ill-behaved nature in this situation, we look at the probability mass *P(f_i _*∈ *V_i _*| 0), and demand it to be above a certain threshold. This avoids the confusion of rarely occurring feature values with rules, since this significantly lowers the probability that all mass ends up with negative points by chance.

In the algorithm we construct below, we select good features to extract rules from, as well as combinations of two feature dimensions. While it is feasible to explore the use of even more features simultaneously in a rule, we abstain from doing so to preserve the balance between exhaustiveness and system performance. The steps to efficiently find these rules are as follows:

1: initialise *R *= [ ], *T *= [ ]

2: **for all ***i ***do **divide the values for *f_i _*into two bins *V_i_, ${\bar{V}}_{i}$*

3: **end for**

4: **for all ***i ***do**

5:   **if ***Count*(*f_i _*∈ *V_i_*, 0) *> threshold ***then**

6:     Add *i *to *T*

7:     **if ***Count*(*f_i _*∈ *V_i_*, 1) = 0 **then**

8:       Add rule (*f_i _*∈ *V_i _*→ 1) to *R*

9:     **end if**

10:   **end if**

11: **end for**

12: **for all ***i*; *j *∈ *T ***do**

13:   **if ***Count*(*f_i _*∈ *V_i_*; *fj *∈ *V_j_*, 1) = 0 **and ***Count*(*f_i _*∈ *V_i_*; *f_j _*∈ *V_j_*, 0) >*threshold ***then**

14:     Add rule (*f_i _*∈ *_i _*∧ *f_j _*∈ *V_j _*→0) to *R*

15:   **end if**

16: **end for**

A few things to note:

• As many of our features can take any real value in the interval [0,1], bins are constructed to re-establish a binary nature, i.e. membership of *V_i _*is analogous to *f_i _*= 0 in the case of bi-valued features. Respectively, *V_i _*designates *f_i _*= 1.

• For the sake of legibility, we implicitly assume *V_i_, V_j _*to be the 'right' bins. In reality, membership to both *V_i _*and ${\bar{V}}_{i}$, respectively *V_j _*and ${\bar{V}}_{j}$ are checked.

• Because *P*(*f_i _*∈ *V_i _*| 0) = *P *(*f_i _*∈ *V_i_*, 0)/*P*(0) and *P*(0) is a constant for a given training set, it is more efficient to work with joint probabilities.

• Because *Count*(*f_i _*∈ *V_i _, f_j _*∈ *Vj*, 0) ≤ min(*Count*(*f_i _*∈ *V_i_*, 0)*, Count*(*f_j _*∈ *V_j_*, 0)), we can already eliminate many combinations of feature dimensions to consider; this is the function of the set *T*. In our experiments, this reduces the number of combinations to check from 3.7 million to 30,000 and keeps the above algorithm tractable.

Important to note is that this algorithm now gives us a tool to also select for negative examples in a distant supervision-like fashion. The basic selection criterion adapted from this general framework relies on the augmented probability of having a positive label, given that the relation exists in the labelled data. As argued before, a similar reasoning generally does not hold for negatives, rendering selection for them infeasible. However, the rules extracted by the above algorithm can serve not only to eliminate very unlikely candidates for positive labelling, as previously done. In fact, because these rules try to encode sufficient conditions for negativity, we can also employ them to distinguish a subset of all the other unlabelled data as being very likely negative. This offers us the opportunity to add both positive and negative points from our unannotated corpus, a technique not feasible in the basic distant learning framework.

The threshold effectively decides the amount of rules extracted from the labelled data, and can be seen as an additional hyperparameter in the model. Based on our dataset, we found a threshold of 20 - 30% of the size of the (annotated) data set to give the most balanced results in terms of precision vs. recall. Depending on the application requirements, a lower threshold will improve precision, while a higher threshold would have us expect an improvement in recall.

## Results and discussion

### Subject and data

The Gene Regulation Network Task tries to accomplish detection of relations overarching a diverse set of molecular interactions. Specifically, six different types of relations are to be extracted: inhibition, activation, requirement, binding, transcription and regulation. The training and development set consists of 134 sentences, jointly encoding 230 interactions. On average this amounts to 38 examples per relation type. Considering the specialised language and grammar often used in scientific publications, the amount of training data seems rather sparse to learn a good general representation in such a complex output space.

As previously argued, this is the main motivation for including additional data for use in the methods described above. We therefore augment the dataset we have with all sentences from PubMed abstracts responding to the query for "*bacillus subtilis sporulation*" (as accessed on 16/08/2013). Beginning from the annotated data points, we add a sentence from those unannotated texts if it contains at least two entities that also occur in our annotated data. Without these entities, a sentence could indeed never encompass a candidate data point for a relation. As such, from the initial 14,109 sentences, only 1,859 are retained, resulting in 11,778 possible entity pairs. Although of minor influence on the end result, we also leave out sentences that are already in the training set. In Table 2 we have shown the average amount of data points that effectively got added to the training set for each system.

**Table 2:** Number of data points from unannotated corpus used in our system, averaged over the six separate SVMs (one per relation type).

System	positives	negatives
[BASIC]	616	0
[PRE-SEL], select POS, no NEG	563 (= 91.3 %)	0
[PRE-SEL], select POS, select NEG	563	9,967 (= 90.6 %)

Also mentioned is the percentage of the respective total candidate pool this is. The positive candidate pool consists of all data points conforming to the distant supervision criterion (# = 616) as seen in the [BASIC] system, while the negative pool is the complement of this (# = 11,162).

### Evaluation

#### The Slot Error Rate

From the predictions, a network gets constructed with the entities as the nodes and the relations between them as arcs. This network is then used for measuring performance: it gets compared to the reference by means of the *Slot Error Rate *(*SER*). This measure is defined by [20] as:

$$SER=\frac{S+I+D}{N}$$

with:

• S the number of substitutions, i.e. edges that are predicted, but with the wrong type;

• *I *the number of insertions (false positives);

• *D *the number of deletions (false negatives);

• *N *the number of arcs in the reference network.

For the following analysis, we further define

• *C *the number of correctly predicted relations;

• *M *the number of arcs in the prediction.

With this notation, precision and recall can be written as:

$$P=\frac{C}{M}=\frac{C}{C+S+I};\;\;\;R=\frac{C}{N}=\frac{C}{C+S+D}$$

The main motivation of [20] in proposing this error measure is the observation that *F_1_*, the often-used harmonic mean of precision and recall, can be seen to be:

$$F_{1}=1-\frac{2S+I+D}{N+M}$$

This derivation leads to believe that substitutions get overweighted in the use of this scoring mechanism. While by no means questioning the usefulness of the separate components (precision and recall), the *SER *gets proposed as a more balanced way of combining them as a means to compare systems.

The devil is however in the details; or rather, the denominator. While it is true that *S *gets a bigger weight in the numerator, one has to account for the weighting of the different components in the denominator, since

N+M=2(C+S)+D+I

where we use that *N *= *C *+ *S *+ *D *and *M *= *C *+ *S *+ *I*. A similar weight scheme can hence be seen in the denominator as well, softening the argumentation against it. With a similar derivation, one finds:

$$SER=\frac{N-C+I}{N}=1-recall+\frac{1}{N}.$$

This insight shows us that in attempting to lower the weight for *S*, this error rate has become completely independent of this factor altogether (since *N *is a constant, given the reference network)! Furthermore, the unboundedness of this measure can be fully attributed to the number of insertions. This can explain the prevalence of conservative systems that this task has received: as can be seen from the official results, all but one submission have a very low number of arcs in their prediction, which could be attributable to pursuing a low *I *figure.

#### Error measures: uses for comparison and model optimisation

By this analysis, we wish by no means to imply that the *SER *is a *bad *scoring mechanism per se. This kind of *word error rates *is widely used in several research branches, and with good reason. However, as the name somewhat shows, these are situations where a more or less fixed number of *slots *need to be 'filled', such as (speech) phoneme recognition or named entity recognition. In our notation, this would be equivalent to M≅N. If this constraint is taken into account, one can show that $SER≅1.5\left(1-F_{1}\right)$, which is exactly what [20] find in their comparative analysis of measures.

In different settings however, where the above approximation is not sure to hold, the choice of *SER *implies an additional degree of freedom, of which the consequences are not evident to grasp. In this more general case, *SER *is seen to overly reward precision in a great part of the result space. This can even occur at the cost of recall, as will be shown below. We believe there is an interesting opportunity for further research and discussion on this matter. Interesting, more general analyses can be found in both [21] and [22]. In the light of this study however, we mainly wish to highlight the inherent bias towards precision this design choice entails. As we are investigating methods of obtaining recall, this is certainly a factor to take into account.

**Figure 3:**
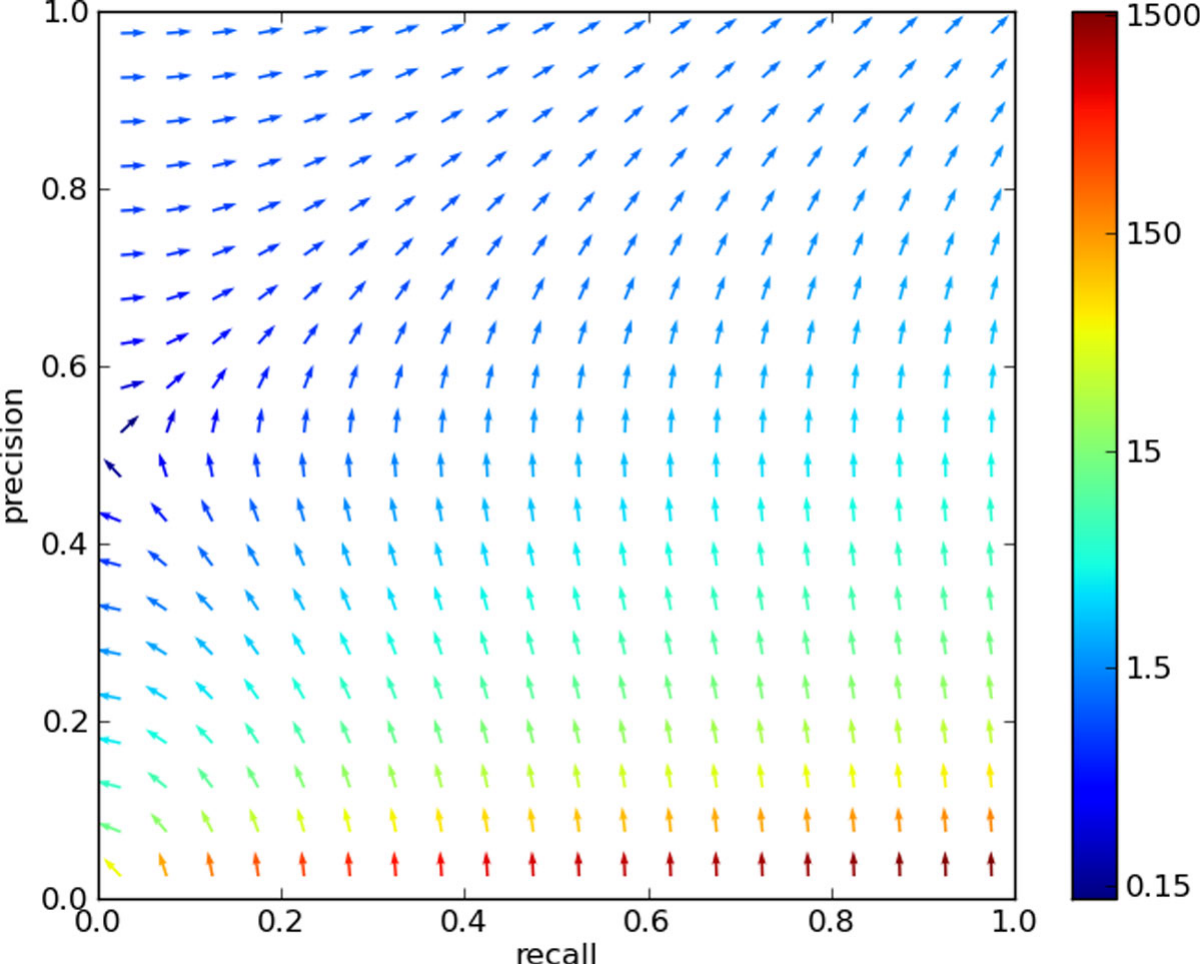
***SER *gradient field, normalised**. The vector field of *SER *gradients in recall-precision space, when there is no change in *S*. The vectors are normalised, with their colour indicating their size, in logarithmic scale. Furthermore their direction is reversed, since this is a minimisation problem, and hence calls for gradient *descent*.

**Figure 4:**
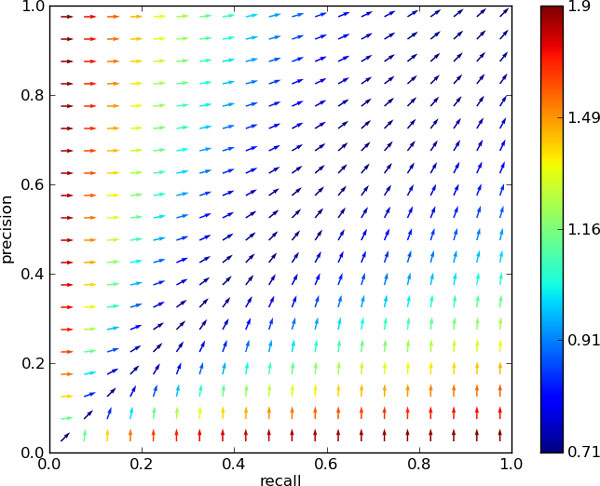
***F*1 gradient field, normalised**. The vector field of *F*1 gradients in recall-precision space. The vectors are normalised, with their colour indicating their size, in logarithmic scale. Of note is the scale difference with Figure 3.

Comparison of performance between different systems (*intersystem *performance) is not the only function of a measure. The same measures get generally used for *intrasystem *measurements as well: in the comparison of multiple incarnations of models, and more commonly, hyperparameter optimisation. In order to asses the behaviour of the latter under different performance measures, we consider an ideally automated setting of optimising, not unlike running a gradient descent/ascent algorithm. In contrast to the case of general convex optimisation however, there is no convergence to a unique optimum. Rather, we are limited by the boundary of our system's performance, generally known as the *precision-recall curve*: the maximum precision that can be obtained for any required recall. Hence, we are driven by the measure's gradient until that border is reached. As we can see in Figure 3, the gradient field of *SER *shows some interesting behaviour. In a substantial region of the recall-precision space, there is an enormous push towards increasing precision. In the region of precision below 50%, this even happens at the cost of maintaining recall. As a result, a system optimised for this measure will generally show good performance, but has little focus on improving recall. For comparison, the analogous field for the *F*_1 _measure is shown in Figure 4, which displays a better balance between favouring recall or precision, based on which is most lacking.

As previously argued, there are use cases where an adequate amount of recall is called for. With this in mind, we point out that *F*_1 _is embedded in a larger family of *F-measures*:

$$F_{\beta }=\left(1+\beta^{2}\right)\frac{PR}{\beta^{2}P+R}$$

where *β *is a measure of the relative value to the end user of recall with respect to precision [23]. We obtain *F*_1 _for the case of *β *= 1, meaning precision and recall are balanced in evaluation. This parameter *β *can be a great tool for the system or task designer to designate the proportion of importance he wishes to place on the precision/recall trade-off. If precision is to be targeted, a value of *β *< 1 will accomplish this, without having gradients go 'against the grain' of increasing both basic measures.

#### Aggregation of predictions and impact on scoring

A final concern is the aggregational processing that occurs before calculating the performance measures. In a traditional machine learning setup, scores are calculated in a *local *scope; meaning, every predicted point is compared to a ground truth, and from the numbers extracted for correct predictions, substitutions, insertions and deletions, the necessary proportions are calculated.

In the GRN task [2], performance is measured in a *global *fashion, due to the processing on the solution set that takes place before calculating the score. This happens in two steps:

• From the predicted classifications a network is built. All scoring is done with respect to this, implying that multiple classifications of a same relation get collapsed into one.

• 'Resolution of redundant arcs': recall that the different types of relations are ordered into a taxonomy (Figure 2). Before scoring, any relation between two entities that is less specific (i.e. higher up the tree) than another appearing in the set, is removed.

We can see that this procedure renders the precision-recall trade-off a lot more intricate than in a traditional machine learning setting. In a *local *scoring procedure, the number of true positives can never decrease by adding more predictions; this is the main logic behind Receiver Operating Characteristic (ROC) curves as monotonously non-decreasing functions. Analogously, in the recall-precision space, this ensures a non-increasing curve of attainable points. Furthermore, this curve spans the whole range of recall: a recall of 100% is always attainable with a precision of at least the ratio of positives in the test set, a worst case that corresponds with classifying all test points as positive (see [24] for a thorough analysis of this and a performance measure that ensues from this, the Area Under Precision Recall Curve (AUCPR)). These principles no longer hold when removing predictions prior to measuring; adding a more specific prediction to an existing true positive renders the latter as non-existent, and recall at the end of the precision-recall curve will be limited by the ratio of positives that have the most specific relation (the leaves of the hierarchical tree in Figure 2). This dynamic stands orthogonal to research on performance measures in a hierarchical setting (as in [25]), which is pursuing less level-dependence in assessing predictions.

This demonstrates that attaining sufficient recall is a greater challenge than in a regular setting. Furthermore, by adding a layer of complexity, it convolutes multiple tools that are basic in systems engineering: error analysis, model selection and comparison. We therefore wish to advocate the addition of *local*, unprocessed evaluation figures in future instalments of this task.

### Experiments

Results for our experiments can be seen in Table 1. Each system has seen its hyperparameters optimised separately by a grid search, 25-fold cross-validated over the training data. The basic method we propose is entry [BASIC] in this table. Even without any added noise-balancing measures, the distant learning framework can already showcase more than a doubling in recall compared to the original submission results of [4]. In light of the previous discussion, this demonstrates a manifest improvement in this dimension. Results for the probabilistic pre-selection approach we developed can be found in the entries under [PRE-SEL]. There we explore several possibilities. In the first (*select POS, no NEG*), we only include (and filter) positives from our unlabelled set, in the fashion of our basic method. The second entry, *select POS, select NEG*, also employs the found rules to further add negatives from the unannotated corpus. Both are able to display a further improvement in F1, while still maintaining a good recall-precision balance. Especially the application of the filter to add negatives (*select POS, select NEG*) warrants a substantial rise in F1 score through an additional improvement in precision compared to the model that only selects positives.

To further evaluate the value of our pre-selection step, we also include the entry *select POS, all NEG*, which includes all negatives without filtering them. Compared to disregarding negatives from the unlabelled set altogether (*select POS, no NEG*), this can be seen as greatly improving precision, but at a sizeable cost to recall. This strengthens the contribution of our algorithm, as the entry *select POS, select NEG *shows an even greater increase in precision without overly damaging recall, leading to an impressive rise in F1.

From Table 2, we can see that this model filters out less than 10% of the potential negatives; this is enough to alleviate the most disruptive noise.

These results confirm the value of filtering the unlabelled data before presenting them to the learning algorithm. As this has been done here exclusively based on the limited amount of labelled data, leveraging additional knowledge in this step could generate even more significant gains.

Weighted regularisation (entry [W_REG]), a method traditionally suggested to handle additional noise in semi-supervised frameworks, also obtains a high recall for this test. This comes however at a very severe cost to its precision, compared to our newly developed solutions. This demonstrates the idiosyncratic nature of our methods as applied to this particular task with respect to mainline distant supervision methods, and further validates their contribution compared to utilising standard approaches.

Deeper study is required on the impact of our methods, since the performance of a system greatly depends on e.g. the features it uses. It remains an open question what the impact is of these implementation choices, such as the feature representation used, data preprocessing, etc. in comparison to the higher-level model choice. We suspect that a more fine-grained encoding of sentence context could further contribute to the performance of any system in this field.

### Related work

In information extraction −and relation extraction in particular− a major bottleneck is the lack of sufficient annotated examples. The manual labelling of enough training instances in order to build an accurate classifier is often prohibitively expensive. On the other hand, collecting a large quantity of unlabelled textual data is cheap. Thus, it is interesting to train the extraction system on a small annotated corpus and in some way improve the quality of the learned classification patterns by exploiting the unlabelled examples. This had lead to bootstrapping, semi-supervised and even unsupervised learning techniques. A good overview on semi-supervised learning, the framework in which this work is embedded, can be found in both [26] and [27].

The oldest methods regard self-training and co-training, where a classifier is trained iteratively. In self-training, examples from the pool of unlabelled instances are chosen in the next training step to which the current classifier assigns labels with most certainty. In co-training, examples are chosen in the next training step to which two or more current classifiers that possibly use an independent feature set assign labels with most certainty [28]. Such a set-up promotes that the newly introduced training examples have similar patterns as the originally labelled examples, so no radical new patterns are learned at least not in the first steps of the iteration. This approach also does not offer an answer to the danger that the obtained classification function drifts away from the real classification boundary. In a variant scenario, a generative probabilistic classifier is used (i.e., probabilities are not estimated directly, rather they are estimated indirectly by invoking Bayes' rule, e.g., a naive Bayes classification) for the training of the initial classifier based on the seed set of labelled examples. The Expectation Maximization (EM) algorithm is then used to train the classifier that learns both from the labelled and unlabelled examples [29], but the algorithm can easily get stuck in a local maximum.

In so-called open domain information extraction, frequently occurring patterns that signal a relation between two entities are identified in a large set of unlabelled data [30,31]. These techniques are not well suited for the extraction of relations in the biomedical domain, especially when the detection of infrequent relations is targeted.

Another line of research is the generation of additional features from the unlabelled data. One recent work is [32], building on the work of [33]. Those methods generally obtain state-of-the-art performance, but fail to improve on them significantly.

The relation extraction models that we present in this paper are closest to the work of [15]. These authors find sentences in Web documents that contain two given entities. It is a priori known that these entities are involved in the sought relation. The selected sentences contain positive as well as negative examples of the sought relation. The negative examples for training the classifier are sentences in Web documents that contain two given entities for whom it is known that the sought relation does not hold between them [14,16] on the other hand approximate this by the equally often-used *closed world assumption*, which dictates that all relations are in the knowledge database. To cope with the noise in the set of positive examples, weighted regularization is used when training a SVM, as we do in this paper. Our experiments on texts from the biomedical literature show that this weighted regularization did not yield the best results for semi-supervised learning. We have proposed a semi-supervised model with probabilistic pre-selection of positive and negative examples from the pool of unlabelled examples that makes use of the knowledge in the labelled examples in a demonstrated effective way in order to select unlabelled examples. This model improves the results of state-of-the-art weighted regularisation techniques.

## Conclusions

We have explored the addition of unlabelled data to increase the recall of our system. However, the noisy nature of this data tends to affect precision negatively. We have designed a pipeline to autonomously counterbalance this effect, based on no additional external knowledge. A promising extension of this method would be to include specialised external knowledge, either injected directly into the feature representation, or in the process of attributing labels to unannotated data. This could prove to be a powerful technique in attaining a more precise overall system. Another interesting approach could be to construct a more extensive pipeline, using one of the more precision-bearing techniques to improve upon our proposed system.

Promising methods in general information extraction make use of language models (e.g., probabilistic models of word distribution) trained on huge amounts of unlabelled examples in order to find valuable replacements of words in the relation patterns or to identify valuable correlated word features used in the classification [12,34,35]. Recent work in biomedical event extraction already touches upon such ideas [32]. This is a path we intend to further explore in future work.

Another particularly interesting approach is showcased by [36], training a classifier jointly on both labelled and unlabelled data. A promising direction could be to apply similar methods to specialised language corpora, such as the biomedical texts explored in the BioNLP tasks.

We argue for the importance of recall in any information extraction task, to serve as a driving force for automated knowledge collection. This study contributes to gaining a deeper insight in the different factors at play in the 2013 BioNLP GRN task with respect to measuring performance, and the interplay of precision and recall in particular. We hope this will spark further discussion and analysis of both task organisation and submitted systems, thus helping this shared task in driving forward the field of biomedical IE.

## Abbreviations

*GRN*: Gene Regulation Network; *IE: *Information Extraction; *NLP*: Natural Language Processing; *SER: *Slot Error Rate; SVM: Support Vector Machine;

• *S *the number of substitutions, i.e. edges that are predicted, but with the wrong type;

• *I *the number of insertions (false positives);

• *D *the number of deletions (false negatives);

• *C *the number of correctly predicted edges;

• *N *the number of arcs in the reference network;

• *M *the number of arcs in the prediction.

## Competing interests

The authors declare that they have no competing interests.

## Authors' contributions

TP is the lead writer and conducted all data processing and analysis, MFM oversaw the study, helped write the manuscript and provided overall guidance. All authors read and approved the final version of the manuscript.

## Declarations

The publication costs of this article were funded by the Research Foundation Flanders (FWO, grant G.0356.12), and KU Leuven (grant BIL/012/008).

This article has been published as part of *BMC Bioinformatics *Volume 16 Supplement 10, 2015: BioNLP Shared Task 2013: Part 1. The full contents of the supplement are available online at http://www.biomedcentral.com/bmcbioinformatics/supplements/16/S10.
